# Nanocellulose, the Green Biopolymer Trending in Pharmaceuticals: A Patent Review

**DOI:** 10.3390/pharmaceutics16010145

**Published:** 2024-01-21

**Authors:** Keth Ribeiro Garcia, Ruy Carlos Ruver Beck, Rosmary Nichele Brandalise, Venina dos Santos, Letícia Scherer Koester

**Affiliations:** 1 Programa de Pós-Graduação em Ciências Farmacêuticas, Faculdade de Farmácia, Universidade Federal do Rio Grande do Sul (UFRGS), Porto Alegre 90610-000, Brazil; k1rgarcia@gmail.com (K.R.G.); ruy.beck@ufrgs.br (R.C.R.B.); 2 Programa de Pós-Graduação em Engenharia de Processos e Tecnologias, Universidade de Caxias do Sul (UCS), Caxias do Sul 95070-560, Brazil; rnbranda@ucs.br (R.N.B.); vsantos2@ucs.br (V.d.S.)

**Keywords:** nanocellulose, nanofibrillar cellulose, drug delivery, pharmaceuticals, excipients, pharmaceutical technology

## Abstract

The use of nanocellulose in pharmaceutics is a trend that has emerged in recent years. Its inherently good mechanical properties, compared to different materials, such as its high tensile strength, high elastic modulus and high porosity, as well as its renewability and biodegradability are driving nanocellulose’s industrial use and innovations. In this sense, this study aims to conduct a search of patents from 2011 to 2023, involving applications of nanocellulose in pharmaceuticals. A patent search was carried out, employing three different patent databases: Patentscope from World Intellectual Property Organization (WIPO); Espacenet; and LENS.ORG. Patents were separated into two main groups, (i) nanocellulose (NC) comprising all its variations and (ii) bacterial nanocellulose (BNC), and classified into five major areas, according to their application. A total of 215 documents was retrieved, of which 179 were referred to the NC group and 36 to the BNC group. The NC group depicted 49.7%, 15.6%, 16.2%, 8.9% and 9.5% of patents as belonging to design and manufacturing, cell culture systems, drug delivery, wound healing and tissue engineering clusters, respectively. The BNC group classified 44.5% of patents as design and manufacturing and 30.6% as drug delivery, as well as 5.6% and 19.4% of patents as wound healing and tissue engineering, respectively. In conclusion, this work compiled and classified patents addressing exclusively the use of nanocellulose in pharmaceuticals, providing information on its current status and trending advancements, considering environmental responsibility and sustainability in materials and products development for a greener upcoming future.

## 1. Introduction

Cellulose is one of the most abundant and renewable biomaterial resources on earth, with an estimated production of 7.5 × 10^10^ tons per year [1], as it is naturally synthesized in plants and produced by some bacteria [2].

Its use in biomass waste is inserted in the concept of circular economy with regard to the four R’s model of how to “reuse”, “recycle” and best “recover” various waste biomass, in addition to establishing waste “reduction” [3].

Cellulosic derivatives such as nanocellulose have been standing out and generating continuous interest because it is a versatile material with high added value, subject to modifications in the chemical structure and/or addition of functional groups [4]. Therefore, nanocellulose-based materials become an advanced plant-derived material that allows its application in areas such as fiber-reinforced composites, electronic templates, biosensors, viscosity modifiers, food packing and drug delivery [5].

Currently, most marketed nanocellulose comes from high-quality fiber material, such as wood pulp or cotton [6]. However, other plant-based materials can be important sources of nanocellulose, such as coconut fiber [7], cotton stalk [8], sugarcane bagasse [9], wheat straw [10] and tobacco stalk [11]. In addition, agro-industrial residues have demonstrated their feasibility and suitability for commercial purposes [3].

Despite the growing interest in nanocellulose, a mapping of patents has not been carried out so far. Academic reviews published so far have addressed the possibilities of using nanocelluloses for drug delivery in transdermal devices [12,13,14,15], as disintegrants or super disintegrants [16], for wound healing [17,18,19] and for injectables [20,21,22]. Furthermore, nanocellulose has also demonstrated its effectiveness in tissue engineering [19,23,24]. The present review explores for the first time the assessment of existing patents concerning nanocellulose applied to pharmaceuticals. Nanocellulose per se is a novel subject and important studies have addressed several knowledge areas, from engineering to medicine. However, it was necessary to conduct data acquisition on patents, in order to understand how far these studies advanced towards a valuable product/process with monetary potential. Indeed, academic research may have different outcomes, depending on the level of innovation, and the result may generate a product with commercial interest or require further study. Several patents stem from over a decade of research, and various findings may lead to different aspects of the studies being filed as patents. The overview could encompass the development of a product, with certain elements of its production process being patented.

Innovation in pharmaceuticals is the backbone of the pharmaceutical industry and must be duly protected by patenting [25]. Ultimately, patents are frequently used as an indicator of the rate of invention, which is a crucial precursor to innovation [26]. A patent is an intellectual property right issued by authorized bodies to inventors to make use of and exploit their inventions for a limited period (usually 20 years) [27].

Filed patents related to the use of nanocellulose in pharmaceuticals have been gradually increasing and the products have demonstrated their participation in the market as important high added value alternatives to traditional products. Therefore, considering the importance and originality of the subject, this study aimed to conduct a patent survey with a 12-year timeframe, covering the applications of nanocellulose in the development of pharmaceuticals.

## 2. Background

### 2.1. Cellulose and Its Different Sources

Cellulose was first discovered and isolated from plants in 1838 by the French chemist Anselm Payen [28]. Almost a century later, around 1928, the Nobel prize winner Norman Haworth studied the composition of carbohydrates and mapped the composition and structure of various forms of sugar, starch, and cellulose, showing that the covalent bond occurs within the molecule and between the glucose units [29]. Two years later, the scientist Hermann Staudinger reported that the cellulose structure constituted glucose units tightly bound together, forming long molecular chains that are covalently linked. This discovery was the origin of polymer science [30,31].

Ever since, cellulose has been considered the most important renewable biopolymer on the planet and, for the manufacturing industry, it is the main source of sustainable material [32].

Cellulose is indeed characterized as a high molecular weight homopolymer of β-1,4-linked anhydro-D-glucose units [2]. These cellulose molecules are packed in the parallel direction with semi-crystalline microfibrils that are held together by inter- and intramolecular hydrogen bonds and by van der Waals forces [5,33].

These microfibrils contain crystalline and amorphous regions. Amorphous regions are present on the surface and are found along the length of the microfibrils [34]. The size of a microfibril varies according to the origin source; for example, the diameter of microfibrils from wood is usually around 4 nm, with a length of a few micrometers [33,35].

### 2.2. Nanocellulose

Nanocellulose was originally introduced by Turbak et al. [36] and Herrick et al. [37]. The researchers first named nanocellulose microfibrillated cellulose (MFC) and demonstrated that a gel-like material was produced by treating wood-based cellulose fiber suspensions with a high-pressure homogenizer. In the mid-1980s, a Sweden company, STFI AB, now Innventia AB, started to work with MFC. The purpose was to use the nanomaterial in paper applications [38].

Over time, many companies became active in the field. The history of microfibrillated cellulose innovation started with three patents filed in 1983—US 4341807 [39]; US 4452721 [40]; and US 4481076 [41]—and two papers [36,37] published by the ITT Rayonnier group. The number of studies grew to 825 by 2007, and today there are more than 5000 patents about MFC filed around the world [42].

Currently, nanocellulose is defined as a cellulosic material that has at least one of its dimensions (diameter, length or width) within the nanometer scale [2,43,44,45,46]. In general, it is a term used to refer to cellulose crystals or fibers having lengths of a few micrometers and diameters between 1 and 100 nm, which can be extracted from natural cellulose fibers [47].

It is considered a pseudoplastic material and exhibits the property of forming gels or fluids that are viscous under normal conditions; however, it eventually flows, when shaken, agitated or otherwise stressed. When the shearing forces are removed, the gel regains much of its original state [48].

The field of nanocelluloses is subdivided into three categories: bacterial nanocellulose, nanocrystalline cellulose and nanofibrillated cellulose [2]. Each type of nanocellulose refers to its production method, as summarized in Table 1.

Table 1 shows the three types of nanocellulose that are obtained, depending on which extraction method is applied. NFC requires a physical method to be used, such as micronizer grinding, microfluidization or high-pressure homogenization, to obtain the delaminated nanofibers. Obtaining NCC requires acid hydrolysis to remove amorphous cellulose. The obtained nanocrystals have a needle shape. BNC, when compared with plant nanocellulose, has a higher purity, due to its obtainment using Gram-negative strains of bacteria such as *Acetobacter*, *Achromobacter*, *Rhizobium*, *Botulinum*, *Pseudomonas*, etc.

Owing to its various different obtention methods and its outstanding features, such as biocompatibility, mechanical strength and tunable surface properties, nanocellulose has become a high-value natural source for groundbreaking applications in distinct areas [3,46,50].

**Figure 1:**
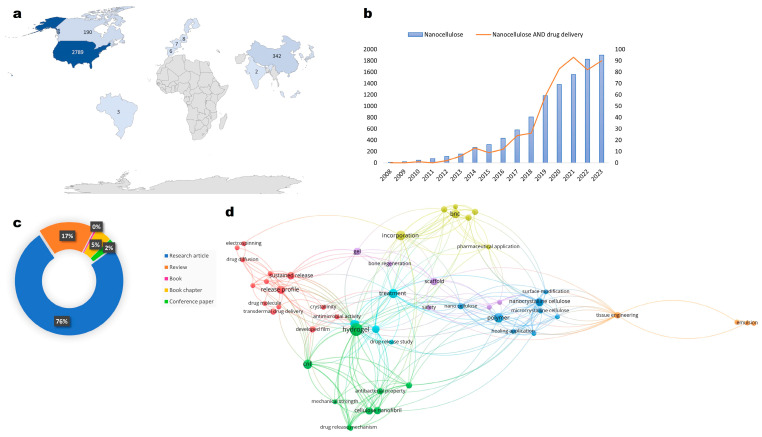
Nanocellulose as a high-value natural source. (**a**) The outstanding global interest in nanocellulose (microfibrillated cellulose) as a study matter. The figures indicated in the map represent the total number of patents referring to nanocellulose in distinguished fields; (**b**) graphic representation of the number of publications per year using the search term “nanocellulose” (blue bars) and the combined terms “nanocellulose AND drug delivery” (orange line) in Scopus database; (**c**) donut chart; and (**d**) bibliometric data concerning the latest articles, book chapters and other publications depicting original research and reviews considering the combination of nanocellulose and drug delivery topics.

All over the world, innovations with nanocellulose feature among the subject matter of patents. The WIPO database presents 14,637 search results for patents related to “microfibrillated cellulose”, the most usual term, since the year 1984. Figure 1a presents the ranking of the top 10 countries with the highest number of patents. Included in this list are 2999 patents filed through the Patent Cooperation Treaty (PCT), which allows the application for patent protection of an invention simultaneously in several countries, through a single deposit called “International Patent Deposit” [27]. Moreover, 1942 patents filed with the European office are considered in this ranking; such interest may be explained as being due to cellulose’s versatility. The biopolymer offers the advantage of the ability to be tailored, to achieve the desired features for application in several fields [1].

Another possible explanation resides in the fact that, in many countries, wood has always been an important part of their livelihood. The main goal of these countries was to make full use of the versatility of this material without causing damage to the environment and the ecosystem. Consequently, investments in expertise and new technologies were necessary. New ways of using wood emerged and, with them, the need to establish innovative strategies for prospective commercial opportunities [51]. Thus, the incorporation of nanocellulose into products and processes boosted its applications in new industrial sectors, including biomedical, environmental, energy, and pharmaceutical sectors [52,53]. In addition, its multifunctionality has been explored by consolidated companies such as Innventia (Sweden), Daicel (Japan), UPM Kymmene and VTT (Finland), Borregaard (Norway), Rettenmaier (Germany), Weidmann (Switzerland) and innovative ones such as GranBio (Brazil) and Finecell (Sweden).

### 2.3. Nanocellulose in Pharmaceutical Applications

Over the last decade, the number of research articles on nanocellulose has increased, showing the important role of this nanomaterial in pharmaceutical technology development. Figure 1b depicts the increase in these publications throughout the years.

In the early years of nanocellulose studies, one of the main challenges concerned accomplishing suitable extraction techniques in different instruments without high energy demands [54]. Over time, many researchers successfully determined an extraction basis and, since then, the rising interest in applying nanocellulose to different areas has settled [46]. The wide applications of nanocellulose and its intrinsic relationship with a pharmaceutical approach are depicted in Figure 1d.

Regarding pharmaceutical applications, the first study displayed by the Scopus database was entitled “Isolation of nanocellulose from pineapple leaf fibers by steam explosion” [55], in which the authors explored the obtention of nanocellulose for biomedical and biotechnological applications, such as wound dressings, drug delivery and tissue engineering.

Sequentially, in 2011, a Finnish research group demonstrated the use of nanocellulose as a filler for tablets [56] and as a matrix for the controlled delivery of drugs [57,58]. Since then, a growing number of studies considering nanocellulose for tablet manufacturing have been undertaken, with nanocellulose as an excipient performing different functions. For instance, nanocellulose has been applied as a disintegrant [59,60] and a super-disintegrant [16]; as a matrix for the controlled delivery of drugs [61,62]; and also incorporated with an inclusion complex as a delivery system [63,64,65]. Furthermore, a transdermal delivery approach [12,13,14,15,66,67,68] has been mentioned with the obtention of a nanocellulose hydrogel.

Hydrogel is the usual attained form of nanocellulose, and its purposes may range from wound healing [17,18,69,70,71] to part of the design for developing injectables [20,21,22]. Moreover, the tissue engineering field has developed promising research regarding nanocellulose [19,23,24,72].

### 2.4. Innovations Regarding Pharmaceutics

**Figure 2:**
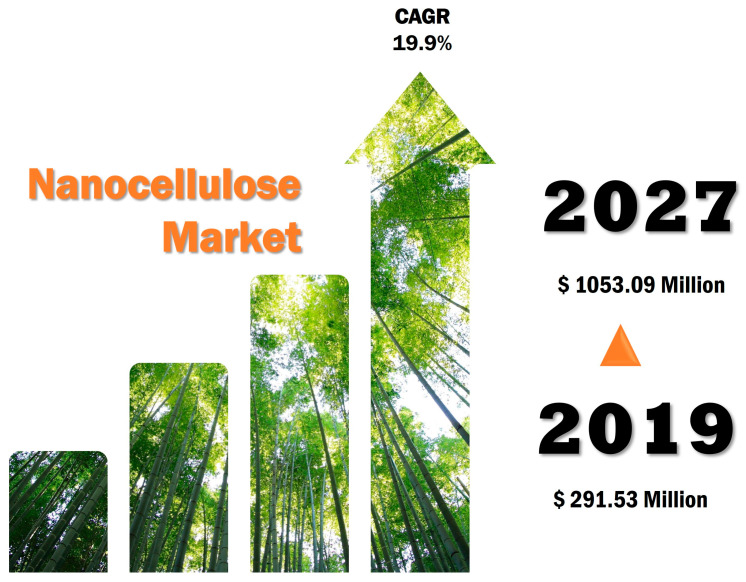
Nanocellulose global market forecast for the period comprising 2019–2027. Data from [74] and picture generated using unplash.com (accessed on 12 January 2022).

The development and industrial application of innovative advanced materials are imperative for technological and economic progress [73]. In accordance with the Global Nanocellulose Fiber Market Report [74], in 2019, the nanocellulose market size was USD 291.53 million, and, by 2027, it is expected to reach USD 1053.09 million, corresponding to a compound annual growth rate (CAGR) of 19.9% during the forecast period (Figure 2). Nonetheless, there are some challenges to overcome, in order to reach economic growth. In fact, the biggest concern lies in standardized production, with attention to the evolution of techniques and environmental care, rather than cost control, which may vary depending on its application and production mode [3].

A few companies have monopolized the successful commercialization of nanocellulose and its high value added products. In 2018, a Swedish company named Cellink AB developed and patented, jointly with UPM-Kymmene, the first bioink designed to print human tissue models for 3D-bioprinting systems. The bioink is composed of alginate and hydrated cellulose nanofibrils that act as a regulator of rheological behavior. The nanofibrillated cellulose dispersion had an extremely high viscosity at zero shear and a viscosity of about 10 Pa·s at 100 s^−1^. That is what contributed to its high printability. The chemical modification of cellulose pulp, prior to mechanical disintegration, gives rise to optically transparent hydrogels, giving it a semi-translucent aspect, and, thus, making it compatible with many advanced imaging techniques. A 5 mL syringe of sterile NFC thickener is commercialized at the price of USD 139.00 [75,76].

Another successful study that evolved from academia to global commercialization is the product GrowDex^®^, launched in 2014 by UPM-Kymmene (Helsinki, Finland), a Finnish company that is often referenced in nanofibrillated cellulose studies. In fact, the product was born as an interdisciplinary effort made by UPM and researchers from the University of Helsinki, in which research was carried out for ten years prior to the product’s entrance into the market. The product is a ready-to-use hydrogel extracted from birch pulp and presents the benefit of easily replacing animal-derived matrices without lot-to-lot variations. It mimics the extracellular matrix for 3D cell culture applications, supports cell growth and differentiation consistently and is commercialized worldwide at the price of EUR 120.00 for 5 mL [77].

A recent evolution of innovation involving nanocellulose was also presented by UPM-Kymmene: the GrowInk^®^. It is a bioink product family developed for 3D printing that was launched in 2020. The bioink is composed of two main components, nanofibrillar cellulose and water, providing a fully defined matrix which can be mixed with cells and customized with additional molecules depending on the cell application. The version containing pure nanofibrillated cellulose is commercialized at EUR 135.00 for 5 mL [78].

These novelties exploit nanocellulose’s structural similarity to extracellular matrices (ECM), in terms of both its porosity and its interconnected framework within the structural hydrogel and the fibrillar arrangement of cellulose that somehow is analogous to collagen and fibronectin in native ECM, not to mention the biocompatibility with cellular activities [79]. The directed efforts made to ensure that research evolves into products with intellectual property signalize the high added value of the nanofibrillated cellulose when compared with its raw material [3].

There is also an increasing interest in cosmetic developments using microfibrillated cellulose (MFC) as a thickening agent, as an alternative to synthetic and imported polymers. In Brazil, the idea came at the time of the SARS-CoV-2 global pandemic, when thickening agents were out of commercialization for alcohol gel production and, consequently, for cosmetics. Subsequently, a partnership between Klabin S.A. and Kind to You Cosmetics has resulted in the use of MFC’s multifunctionality to develop cosmetics with the premise of a creamier texture and more intense skin hydration. The partnership uses microfibrillated cellulose derived from paper manufacturing (US 2021/0197540 A1) [80] and promotes a more sustainable product.

## 3. Patent Survey

In order to assess the current status of innovation regarding nanocellulose in pharmaceutical applications, a patent search was carried out, employing three patent databases: WIPO (https://www.wipo.int/patentscope/en/, accessed on 7 September 2023); Espacenet (worldwide.espacenet.com, accessed on 6 September 2023); and LENS.ORG (www.lens.org, accessed on 10 September 2023). The search consisted of a patent survey, sorted by priority date, over a timeframe of 12 years (from 2011 to August 2023). A patent query was performed by applying the terms “nanofibrillar cellulose”, since products are already being commercialized using this terminology, “nanofibrillated cellulose”, and its synonyms “microfibrillated cellulose”, “cellulose nanofibers”, “cellulose nanocrystals”, “nanocrystalline cellulose”, the classical and general term “nanocellulose”, which comprises a plethora of applications, and “bacterial nanocellulose”, which, in this search, was considered a separated niche, due to its non-plant-based source. These terms were combined with words like “pharmaceutical”, “excipient” and “drug delivery”, with the Boolean operator “AND” and the truncation symbol (*) in title or abstract.

## 4. Results

There were a total of 1952 patents that included the search terms. In order to facilitate the presentation of the results, the documents were divided into two main groups: (1) NC, referring to the sum of results obtained with the interchangeable terms “nanocellulose”, “nanofibrillar cellulose”, “nanofibrillated cellulose”, “microfibrillated cellulose”, “cellulose nanofibers”, “cellulose nanocrystalline” and/or “nanocrystalline cellulose”, and (2) BNC, referring to bacterial nanocellulose. 

**Figure 3:**
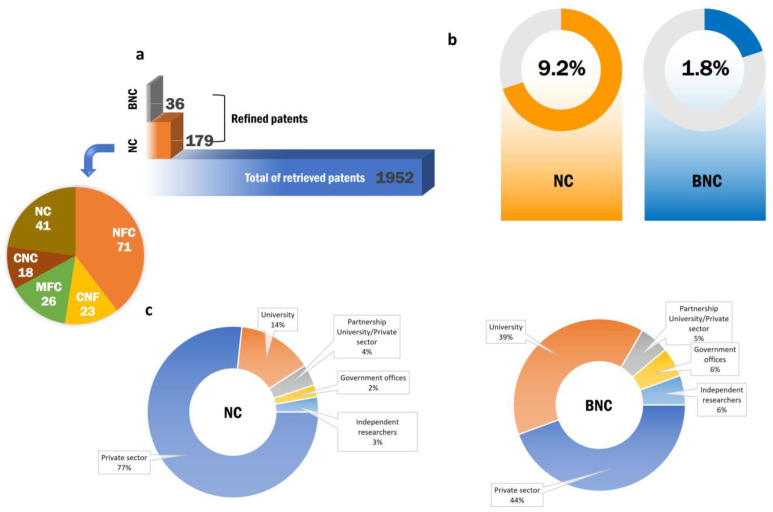
Results of search terms. (**a**) total number of patent documents in each group, termed “nanocellulose” (NC) and “bacterial nanocellulose” (BNC). The NC group represents the patents in the pharmaceutical field and the BNC group represents the patents in the pharmaceutical field, excluding duplicates. (**b**) Representative percentage of refined patent documents including the terms “nanofibrillar cellulose”, “nanofibrillated cellulose”, “microfibrillated cellulose”, “cellulose nanofibers”, “cellulose nanocrystalline”, “nanocrystalline cellulose”, “nanocellulose” and “bacterial nanocellulose”, combined with a Boolean operator, truncation symbol and “pharmaceutical”, “excipient” or “drug delivery”. (**c**) Donut chart representing the percentage of patents referring to the search terms aforementioned.

Excluding the duplicates and selecting only the documents referring to the use of the nanomaterials’ extraction, development and application in the pharmaceutical field, 179 results including the terms compiled in the NC group were retrieved over the cited lapse time, which represent 9.2% of the included terms. The remaining 1.8% (n = 36) corresponded with the term “bacterial nanocellulose (BNC)” (Figure 3).

To better visualize the types of patent applications involving nanocellulose in pharmaceutics, the holders of these patents are depicted in Figure 3c. From the evaluated documents, 76.7% were from the private sector, mainly headed by companies that have a history of working with cellulose. Universities were responsible for 14.2% of granted patents and their interests vary from design and manufacturing to drug delivery. The research that originated from the partnership between the private sector and universities represented 4% of patents, 2.3% of patents came from government offices and 2.8% was from independent researchers. Furthermore, the nanocellulose market growth also reflects the presented figures for bacterial nanocellulose (BNC), patent filings of which have been rising mostly because of academic contributions, which are responsible for 38.9% of the documents. The private sector still achieves a bigger part, comprising 44.4% of innovations. The other sectors comprise a market share of 5.5% each.

**Figure 4:**
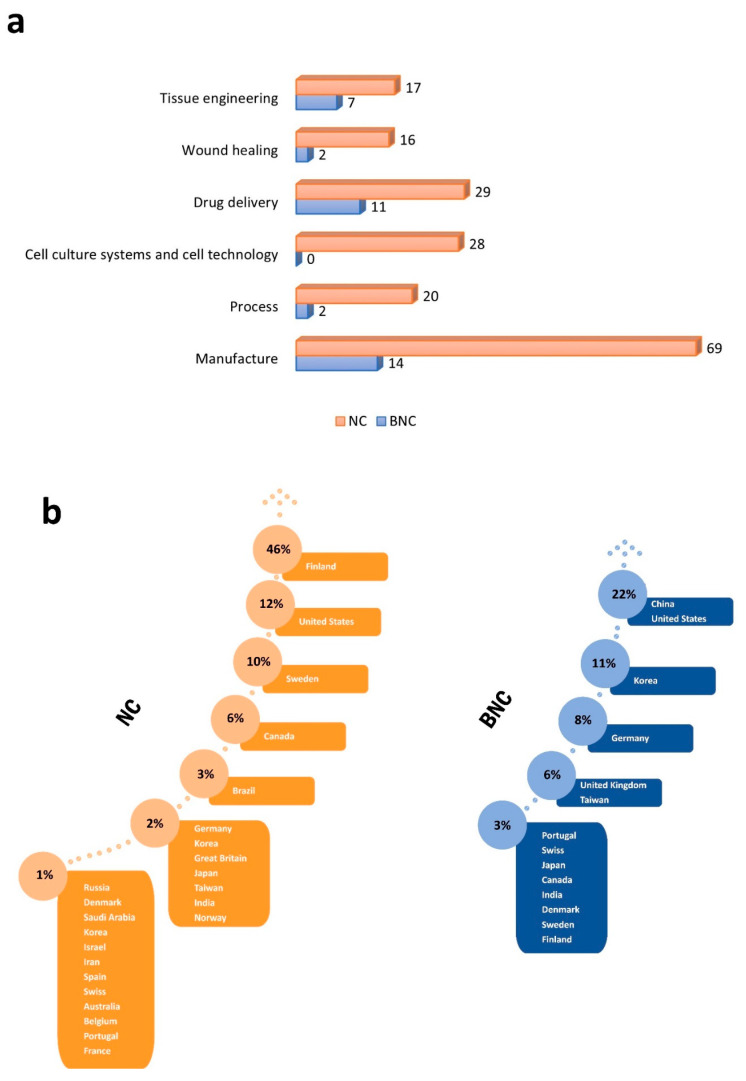
Patents divided by clusters. (**a**) Relative number of patents classified in each cluster. (**b**) Representative percentage of applicant countries of patents regarding the joint search results for the terms “nanocellulose” (NC), “nanofibrillar cellulose”, “nanofibrillated cellulose”, “microfibrillated cellulose”, “cellulose nanofibers”, “cellulose nanocrystals” and/or “nanocrystalline cellulose”, and “bacterial nanocellulose” (BNC).

In view of the different application purposes and the multiple ways of manufacturing nanocellulose for use in pharmaceutics, the present manuscript selected and divided the patents (Figure 4a) into five major clusters: (1) design and manufacturing, subdivided into manufacturing and processes for nanocellulose conception; (2) cell culture systems and cell technology; (3) drug delivery, classified according to release site and/or system; (4) wound healing; and (5) tissue engineering. The set of patents published in the last 12 years regarding the uses of nanocellulose in the pharmaceutical field is depicted in Figure 4.

The countries of the applicants and the corresponding percentage of patents filed for each term, as well as for BNC are shown in Figure 4b.

The complete list of patent numbers is shown in the supplemental material (Tables S1 and S2). It is important to highlight that the total number of patents described herein and divided into clusters comprises all patents’ jurisdictions, but only a document regarding each applicant was accounted for. 

Regarding the NC group, the country that invests the most in innovations is Finland, followed by Sweden and United States. These are the home countries of traditional wood and cellulose working enterprises that have extensive knowledge of cellulose and its derivatives. To date, the insertion of a biomedical sector inside these companies to research and develop plant-based bioproducts seems to be a trend. On the other hand, companies from China were the ones who patented the most applications for BNC. The country is historically a great generator of innovations and stands out in different achievements. Despite its vast territory, the country does not have a tradition of wood manufacturing, which appears to be a barrier in the research of nanocellulose.

### 4.1. Nanocellulose Patents

**Figure 5:**
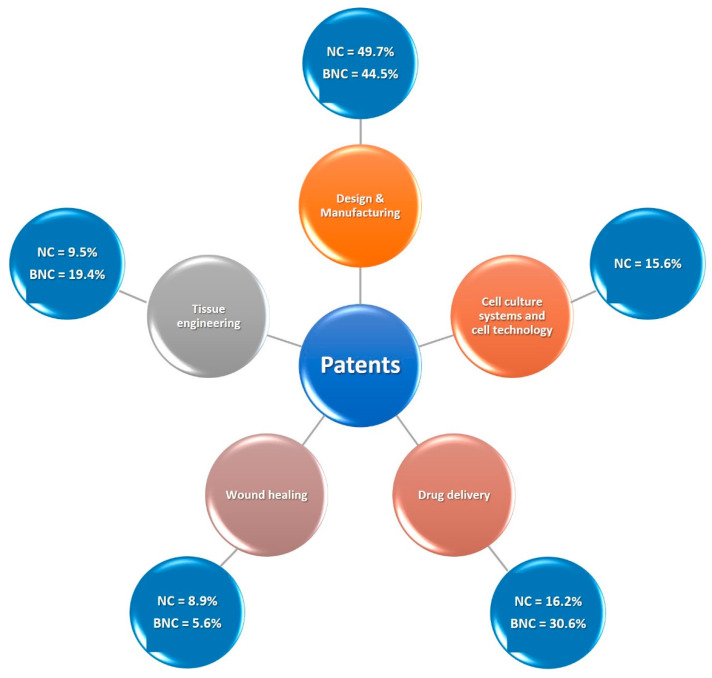
Nanocellulose patents classified into clusters.

Nanocellulose patents were divided into five clusters, which are depicted in Figure 5.

#### 4.1.1. Design and Manufacturing Cluster

The conception of a pharmaceutical product begins with designing its composition and manufacturing the raw material. The following discussion regards the data depicted in Figure 4a. In the pharmaceutical field, every single protected innovation matters; therefore, herein, we are going to present some interesting patents from each cluster. 

The major cluster that discloses the procedures and treatments for nanocellulose was “Design and manufacturing”, presenting 69 patents. U.S. Patent Nº 2016/0184438 A1 [81], for example, provides a process for producing pharmaceutical excipients from lignocellulosic biomass. Lignocellulosic biomass usually results in agro-industrial residues and other plant-based high value-added materials [82,83]. Nevertheless, the main concern of nanocellulose extraction is obtaining a multifunctional product and, at the same time, preserving the environment during the process [84]. Independently of the source, wood-based or plant-based, most lignocellulosic frameworks contain 30–50% of cellulose, 15–35% of hemicellulose and 10–20% of lignin [85], which are attractive options for bulking raw materials in certain fields.

In the aforementioned patent, lignocellulosic biomass was used to produce what is called crystalline cellulose, but may also be referred to nanocellulose and its synonymous terminology, as a pharmaceutical excipient that may function as an anti-adherent, a binder, a coating, a disintegrant, a lubricant, a glidant, a sorbent, a preservative, or another component present in solid dosage forms.

Also regarding pharmaceutical composition, U.S. Patent Nº 2021/0077403 A1 [86] refers to a “Method for preparing pharmaceutical composition and pharmaceutical composition”, which discusses the use of nanostructured cellulose to enhance the bioavailability of poorly water-soluble drugs. The pharmaceutical compound is suitable for different types of administration, especially when it is designed to be composed in the form of a hydrogel.

When the term “hydrogel” appeared, a long sequence of patents showed the high value of nanofibrillar cellulose in this form. Hydrogel is the most usual form in which a product is obtained, after the grinding or homogenization process of nanofibrillar cellulose [87]. It is cited in the majority of patents as a versatile material, due to the presence of a network of interconnected pores allowing the retention of a large number of compounds ranging from drugs to cosmetics for skin treatment. For example, WO 2016/193548 A1 [88] relates to a process for producing sterile nanofibrillar cellulose hydrogel for use in various applications; WO 2018/108341 A1 [89] describes a method for freeze-drying cells in a nanofibrillar cellulose hydrogel; WO 2018/109281 A1 [90] refers to a method for drying nanofibrillar cellulose hydrogel; and WO 2016/102766 A1 [91] discusses the treatment of nanofibrillar cellulose hydrogel at elevated temperatures. Although the documents give the intended use of the hydrogel, the cited patents were classified accordingly into suitable clusters regarding the field of the invention.

Following cluster classification, the “Process” subdivision of “Design and manufacturing” included 20 patents regarding some kind of nanocellulose structure functionalization. In this sense, WO 2012/152997 A1 [92] discusses a coating derived from spray-dried nanocellulose particles for the creation of superhydrophobic surfaces, enabling water repellence in the production of membranes or films. This hydrophobization may occur during and/or after the addition of the nanocellulose particles with the use of a modifier compound.

A functionalization process was also invented and documented by US 2016/0130368 A1 [93] regarding the “Synthesis of nanostructured carboxycelluloses from non-wood cellulose”. The invention comprises an improved process for the preparation of oxidized/carboxy nanostructured cellulose, derived from sugarcane bagasse or cotton cellulose. The patent provides a pharmaceutical composition for microbial treatments by using 6-carboxy cellulose and 2,3,6-tricarboxycellulose, alone or in association with pharmaceutically acceptable carriers or excipients.

#### 4.1.2. Cell Culture Systems and Cell Technology Cluster

The use of nanocellulose for “Cell culture systems and cell technology” was also reported as a protected invention. This cluster included 28 patents concerning the topic. An example of this application is provided by the patent EP 3,750,982 A1 [94], which discusses the improvement of a cell culture plate by using nanofibrillar cellulose as a barrier to divide the cell culture on the plate into at least two compartments. The invention reports the ideal barrier properties provided by nanofibrillar cellulose, preventing cells from passing through the barrier but allowing small molecules or extracellular components to pass. 

Another example is provided by US 2021/0214510 A1 [95], in which a nanofibrillar cellulose hydrogel is used as a three-dimensional matrix for 3D cell culture. The matrix allows cell growth without the interference of growth factors or other components that are usually present in ordinary matrices and affect adherence, differentiation or even cell growth. 

One recently protected invention, US 2020/0164103 A1 [75], refers to the use of the water dispersion of cellulose nanofibrils as bioink, to be used in 3D bioprinting technology. This invention is suitable for 3D cell culturing and for growing living tissues and organs. The applicants claim that this bioink is cytocompatible and, therefore, can be combined with living cells. As mentioned before, this invention has already been commercialized as Cellink^TM^ Nanofibrillated Cellulose, granting a market share to the nanomaterial.

#### 4.1.3. Drug Delivery Cluster

Following the cluster division, the topic “Drug delivery” was linked to 29 patents. The inventions ranged from oral to transdermal delivery. Some of those inventions are summarized as follows. The patent WO 2013/009253 A1 [96] reports the development of films of nanocellulose for the controlled release of drugs. Films for controlled release are made of an insoluble film-forming polymer and a pore-forming agent. In such a case, nanocellulose provides the water insoluble film-forming feature, and another cellulose derivative, such as Hypromellose (HPMC), is responsible for the pore formation. This strategy enables the modification of the permeability of the film and, therefore, of the release control. The film may be used as a coating for solid dosage forms. 

WO 2012/056111 A2 [97], for instance, discusses cellulose nanofibers in a hydrogel matrix designed for drug delivery. The invention may be suitable for intramuscular, intraocular, subcutaneous or topical preparations. In addition, WO 2013/072563 A1 [98] relates to the invention of a drug delivery matrix for the sustained delivery of bioactive agents. The matrix comprises nanofibrillated cellulose, and four different obtention methods are described. Method A comprises the blending of bioactive agents with an aqueous suspension or dispersion of nanofibrillated cellulose (NFC); Method B refers to dissolving the bioactive agents in a solvent or buffer solution to obtain a solution that will be blended with the aqueous suspension or dispersion of NFC and then spray-dried; Method C involves the steps mentioned in Method B with the additional step of element extraction using an organic agent miscible with water, and then the drying step; and, finally, Method D relates to the introduction of an aqueous suspension or dispersion of NFC and extracting some elements according to the procedure described in Method C, which comprise bioactive agents for a further drying process. Altogether, the methods described are intended for the development of intrauterine devices or subcutaneous implants.

Within the advancements in health care, pharmaceutical formulations also boosted research and developments of products intended for injection. The invention US 2021/0085602 A1 [99] concerns an injectable and implantable pharmaceutical formulation, capable of providing a sustained release of one or more active pharmaceutical ingredients. Therefore, nanofibrillated cellulose has been demonstrated to have an ability to be used as a matrix material providing modified release. The matrix comprises nanofibrillar cellulose hydrogel with a fibrillar content ranging from 1 to 8% (*w*/*w*), administered by a syringe. Due to its good stability and bioavailability and its sustained release properties, nanofibrillar cellulose was demonstrated to be a suitable material for subcutaneous, intradermal or intramuscular administration.

A buccal delivery was described In WO 2018/152627 A1 [100], which discusses an artificial saliva gel to treat or ameliorate xerostomia. The gel can comprise a synthetic polymer or a combination of synthetic polymers. In addition, a second polymer, such as a natural polymer, for example, nanocrystalline cellulose (NCC), can be added or replace the carbomer according, to the desired function such as thickening, gelling, or enhancing viscosity. 

Some interesting purposes were also found regarding topical applications, such as WO 2020/031186 A1 [101], which refers to topical formulations comprising cellulose-based materials such as cellulose nanocrystals. The document states that most allergens have a binding site for cellulose and, by applying a nanocellulose material to a skin region exposed to the allergen, the competitive binding of the nanocellulose material to expansin-like proteins is exploited to block or reduce the immune response to the allergen. Furthermore, US 2020/0188538 A1 [102] describes an ultrasound gel for use in internal and oral ultrasound imaging and/or therapy. The gel is also formed using NCC, due to its capacity to crosslink and easily disperse in water. In this case, NCC is used as an additional gelling agent in carbomer-based ultrasound gels, as it can increase the viscosity of the resulting gel, while maintaining its appropriate acoustic properties, as well as a capability to maintain a high viscosity while undergoing gamma radiation sterilization.

Still referring to dermal applications, US 2016/0186377 A1 [103] discusses a hydrated, nanocellulose nonwoven sheet and a method for manufacturing the nanocellulose sheet, which has dermatologically active ingredients. The sheet is claimed to be a device which is capable of transpiring or evaporating water through a dermatological mask, thereby causing a dynamic fluid system between the skin beneath the sheet and the sheet itself. Additionally, there is a method of manufacturing discussed, which is capable of incorporating particulates and solution-based active ingredients at many different phases of the method, allowing for evenly dispersed ingredients.

#### 4.1.4. Wound Healing Cluster

The “Wound healing” cluster included 16 patents regarding the subject. In this sense, U.S. Patent Nº 2021/0030919 A1 [104] refers to a medical hydrogel, comprising nanofibrillar cellulose, for treating wounds by inducing vascularization. The invention reported that nanofibrillar hydrogel can induce vascularization in deep tissues, such as dermis or adipose tissue, speeding up the wound healing process. The product presents the advantage of being applied and easily removed without harm or injuries, as well as protecting the wound from infections and providing a moist environment, which is ideal for the healing process.

Moreover, WO 2018/130390 A1 [105] discusses an invention based on the loading of a hydrophilic natural three-dimensional network, made of cellulose nanofibers, with lipid-based dermal carrier systems. The invention assures that lipophilic active substances can be incorporated into the nanofibrillar hydrogel and can release its contents into wounds, burns and foot ulcers. Its application is not restricted to wound treatment, as it may be widened to the cosmetics field. 

Formulations for wound healing were described by EP 3402462 B1 [106]. The invention refers to the development and use of nanocrystalline cellulose from biological plant material (*Syzygium cumini*), impregnated with nanosilver, in an antibacterial wound care product.

Furthermore, nanocellulose is still being studied as a hemostatic material. The U.S. Patent Nº 2016/0325011 A1 [107] describes the invention of a material in the form of a liquid or a spray mist, for application on a bleeding wound’s surface, that exhibits an excellent solidifying action on all of the plasma, serum and blood and achieves a hemostatic effect quickly and easily. This is an aqueous dispersion, comprising insoluble polysaccharides with an average fiber diameter of 0.001 to 100 μm and a ratio (L/D) of an average fiber length (L) to an average fiber diameter (D) of 5 to 500. These measurement parameters permit the authors to conclude that the polysaccharide in question belongs to the nanocellulose field. The document reported that insoluble polysaccharides are selected from a group consisting of cellulose fibers and chitin fibers, reinforcing this thesis.

#### 4.1.5. Tissue Engineering Cluster

The last cluster classified in the present work is “Tissue engineering”, which is responsible for 17 patents in the timeframe of interest. The U.S. Patent Nº 11103617 B1 [108] refers to the production and obtention of a scaffold of hollow fibers, comprising a mixture of polylactic acid (PLA) and polybutylene succinate (PBS) within cellulose nanofibers (CNF). The invention produces scaffolds for tissue engineering by using the electrospinning process, a technique capable of originating products that mimic a native extracellular matrix. The patent comprises a reinforced CNF with a PLA/PBS scaffold structure suitable for tissue regeneration and vascular tissue engineering.

Another example intended for tissue engineering applications is US 2019/0015550 A1 [109]. The invention refers to the development of a method for preparing injectable thermosensitive cellulose nanofiber hydrogel with the functionalization of its molecular structure. The product showed excellent cell proliferation and bone regeneration. Additionally, the hydrogel exhibited an interconnected structure, enabling it to rapidly gelatinize in vivo, based on its thermosensitivity when undergoing the sol–gel transition temperature. This process facilitated bone regeneration upon implantation in vivo.

Three-dimensional printing of human tissues is also a protected invention regarding the use of nanocellulose. WO 2019/122351 A1 [110] refers to the use of extracellular matrix material combined with biocompatible bioink to bioprint human tissues and scaffolds. The invention is supported by the possibility of using a human tissue-specific extracellular matrix, associated with a polysaccharide hydrogel-based, in this case, nanocellulose-based bioink for 3D bioprinting. The combination of nanocellulose-based biomaterial, together with human extracellular matrix materials, showed an improvement in cell function, viability and engraftment, compared to the nanocellulose-only printed tissues.

### 4.2. Bacterial Nanocellulose Patents

Finally, there are an number of interesting patents concerning bacterial nanocellulose (BNC), which, as mentioned before, was considered in this paper as a class apart. In Figure 3c, it is possible to observe the growing number of patents focused on the subject. Moreover, the application of such a nanomaterial in pharmaceutical research has demonstrated technological improvements and is an alternative to well-established materials. The results of the refined patent search are exemplified as follows.

#### 4.2.1. Design and Manufacturing Cluster

The “Design and manufacturing” cluster was the first one with more patents regarding BNC applications in pharmaceutics, presenting a total of 14 documents. Bacterial nanocellulose has also been studied to create enclosure particle devices, known as pharmaceutical capsules. The patent CN 109223727 A [111] discusses a preparation method of a bacterial cellulose hollow capsule, for filling with pharmaceutical ingredients. The inventors claimed that the production process is simple, with a reasonably low cost, safe and reliable. The process had shown to be suitable for scale-up and mass production. Moreover, the Japanese Patent Nº 2021065224 A [112] is an invention referring to methods to produce capsules made from bacterial cellulose film, which is capable of enclosing particles, and pharmaceutical compositions containing the bacterial cellulose film capsules.

On the other hand, WO 2020/136629 A1 [113] refers to development of BNC powder, which is useful for medicine, cosmetics, food and polymer composites, among others. The invention is a powdered and rehydratable bacterial cellulose and discloses different manufacturing conditions.

#### 4.2.2. Drug Delivery Cluster

According to the divided cluster approach, “Drug delivery” was the classification responsible for the second highest number of patents in this class, encompassing 11 documents. The discussion of some of these inventions is summarized as follows. 

For instance, WO 2011/079380 A1 [114] refers to bacterial nanocellulose hydrogel and a method for producing transparent hydrogels with improved mechanical properties and water content. The authors have envisioned uses for the hydrogel including the production of ophthalmic devices, such as intraocular lenses and contact lenses. The invention can provide drug delivery through the incorporation of drugs and active substances into the BNC hydrogel.

A transdermal delivery approach was invented by CN 106074458 A [115], in which a composite structure of bacterial cellulose and polyacrylonitrile medicine-carrying fiber was prepared and incorporated with an anti-inflammatory agent and a skin penetration enhancer. This composition provided slow drug release for 10 days, creating a possible use as an external dressing for inflammatory issues.

Furthermore, oral delivery was described in the patent US 2012/0308649 A1 [116], where the invention takes advantage of the entangled fibers network originated by bacterial nanocellulose to use it as a matrix to deliver functional components such as drugs, probiotics or nutrients, encapsulated in calcium alginate capsules.

Topical application was approached by CN 210673549 U [117], which describes an invention relating to a bacterial cellulose medical cold compress patch. The inventors assure the reader that the compressed patch is convenient to carry, easy to use, has a good adhesion effect, is not prone to falling off, capable of achieving lasting cooling without active pharmaceutical ingredients, safe in effect, has good biocompatibility and degradability and is easy to produce on an assembly line in a large scale.

#### 4.2.3. Tissue Engineering Cluster

“Tissue engineering” ranked as the third cluster, with seven patents accounted. In this regard, WO 2016/174104 A1 [118] relates to a composite comprising bacterial nanocellulose and sensor- or signal-processing molecules for chip card technology and material engineering. The bacterial nanocellulose composite comprises DNA or RNA or modified nucleotides, together with chip card components intended for muscle stimulation, skin stimulus conduction and neuro transplants, among others.

Tissue engineering is also a protected invention described in US 10767205 B2 [119], where bacterial nanocellulose is used and has its structure modified, in order to produce exopolysaccharides susceptible to hydrolysis and, hence, are bioresorbable and biodegradable by the human organism. This invention is destined to be used as a bioresorbable implant for soft tissue repair.

#### 4.2.4. Wound Healing Cluster

The importance of bacterial cellulose is evidenced by its frequent use in skin repair treatments, in the cases of burns, wounds and ulcers. This material is important to accelerate the epithelization process and avoid infections [120]. Thus, “Wound healing” was also a subject covered in patent research, resulting in two patents. Bacterial cellulose has been used due to its high water uptake capacity and purity. The U.S. Patent Nº 2016/0074520 A1 [121] relates to compositions comprising cellulose hydrogel membranes that are useful in treating wounds, including ocular wounds. The invention is based on the fact that cellulose hydrogel membranes can be synthesized to have a combination of desirable properties, including transparency, strength, and biocompatibility, which make them particularly advantageous for wound healing applications, including ophthalmic wound healing applications. The document safeguards the commercially available trademarks of bacterial cellulose as XCell, Biofill, Bioprocess and Dermafill as possible ways to produce cellulose hydrogel membranes. The other document concerning wound protection is CN 111265709 A [122], in which the invention provides a wound protection film for the prevention of pneumonia virus infection. In such a case, the film is composed of bacterial cellulose and impregnated with a triazole nucleoside solution. According to the inventors, the wound protection film prepared by the invention not only has excellent antibacterial and pneumonia virus infection resistance, but also has good biocompatibility, biodegradability and mechanical properties.

## 5. Discussion

The relative number of patents involving the different types of nanocellulose corresponds to the huge amount of research and publications depicted in Figure 1c,d. The different applications of nanocellulose regarding pharmaceuticals have arisen as an interesting alternative to synthetic compounds and have established a new opportunity for the development of biocompatible, bioresorbable, and biodegradable products. 

There is a consensus that nanocellulose has been used in biomedical applications in general due to its properties of a low cost, easy availability, biocompatibility, good mechanical properties and low cytotoxicity. It was noticed that NFC and NCC are most applied as an alternative to nanofillers, to provide more resistance in drug delivery applications, while BNC has been commercialized for wound-healing treatments and skin-related issues [123].

At the current time, it is imperative to consider environmental responsibility and sustainability in material development. That is why there are so many studies looking at materials from renewable sources that are biodegradable and have highly valuable costs. In this context, nanocellulose has drawn attention due to its characteristics of low density, reactive surfaces, biodegradability, renewability, biocompatibility, being easy to find (as it is derived from agro-industrial wastes) [124] and low toxicity [125].

The majority of patents cover the manufacturing steps to use in order to obtain a high value-added product. Furthermore, the invention protection process of pharmaceutical products has also patented nanocellulose as a drug delivery component, which has been shown to be effective in different administration routes. 

In fact, when a patent is developed, confidentiality means that the reasons that led a certain material characteristic to be turned into a product are not disclosed. Some of these features were, however, presented in this review and discussed, if possible, in Section 2.4. 

Hydrogels are mostly produced with NFC because of its gel-like behavior, due to the highly entangled network of fibrous particles that usually promotes colloidal suspension behavior when diluted and, as gels, when more concentrated [126]. Wound healing bio-adhesives [127], contact lenses [128] and artificial skin [129] are some examples of materials made with hydrogels. In this sense, nanocellulose acts as a gel viscosity enhancer. Depending on the type and concentration of nanocellulose used, the hydrogel can show more elasticity or form linked networks.

In pharmaceuticals, hydrogels focused on nanotechnology are the pioneers among the primary biomaterials developed for human uses. Hydrogels have a number of characteristics that play an important role in their application as biomaterials. They mimic the extracellular matrix’s (ECM) behavior in organs and tissues, due to their rubbery and soft structure in the swelled condition. Hydrogels are suitable for pharmaceutical and biomedical applications because of these aforementioned characteristics, as well as their mucoadhesive nature, swelling and deswelling traits and elasticity [125]. 

Additionally, this review sheds light on the possibility of using nanocellulose as a printable bioink to develop tissues, organs, extracellular matrices, scaffolds and matrices for drug delivery. Thus, reusing agricultural waste or benefiting from the paper production process turns nanocellulose a “green” material, which is important for the upcoming future of pharmaceuticals. 

Cell culture systems and cell technology are the utmost areas of interest for companies that filed several patents regarding 3D cell culture and 3D bioprinting. Some companies have launched nanocellulose-derived products and have commercialized them, reinforcing the concept of it being a high value added product. Therefore, this field seems likely to further advance in the upcoming years. On the other hand, some patents focused on bone or tissue repair, mixing strategies of cell culturing with tissue engineering concepts, thus providing nanomaterial absorbance in the organism. In this sense, nanocellulose has proven to be a fundamental part of the positive outcomes, since the nanomaterial did not jeopardize human functions. Moreover, nanocellulose has demonstrated its importance in wound recovery and has established its use, mainly with bacterial nanocellulose. 

Undoubtedly, nanocellulose has plenty of potential to be part of a greener future of pharmaceutical technology. This is because it is a versatile material that has already been shown to have a high added value when used in the pharmaceutical field. Furthermore, bacterial nanocellulose has proven to be a promising candidate in a range of biomedical applications, due to its high in vivo biocompatibility [130], purity [131], porosity and flexibility, as well as the absence of hemicellulose and lignin [132]. Bacterial nanocellulose also has a great capacity to retain water, which makes it suitable for hydrophilic ingredients [133]. All these aspects contribute to market growth and assure the participation of progressing nanocellulose-based materials in the upcoming future of nanoscience.

Nanocellulose was shown as a green byproduct in the concept of the circular economy, and therefore was sustainable prior to its products’ conception. It can ensure forests preservation and provide a novel destination for agro-industrial residues, which guarantees the application of nanocellulose as an eco-friendly biomaterial.

## 6. Conclusions

Research and investment in the development of products using nanocellulose, regardless of its origin, is a remarkable accomplishment of science and society. This study has shown results that portray the current reality of the scientific community and especially of companies that found in nanocellulose a high value-added material, which was versatile, with a low manufacturing cost. It was possible to map and evaluate a panorama of the last 12 years (2011–August 2023) of technological strategies for the development of nanocellulose products. The major applications were provided, and the inventions were divided into clusters of interest. With the present data, it was possible to comprehend how fast the nanocellulose market is advancing towards greener technology and how suitable this biopolymer is for several applications. Nonetheless, the present work contributes to the nanocellulose field, since it could assist in further decision-making processes for the research and development of pharmaceutical products, boosting the upcoming innovations.

## 7. Challenges and Future Perspectives

It is a fact that, in the field of nanocellulose, the main concern is to obtain a multifunctional product that preserves the environment and explores new ways of addressing sustainability throughout the product’s life cycle. At the same time, depending on the nanocellulose applications, the process of standardization reveals itself to be a question to be answered over time. Nanocellulose per se has been shown as having a high added value when used in pharmaceutical products. Recently, the positive results regarding drug delivery systems have demonstrated the importance and interest of academia and companies in investing in the development of products, which brings with it the concern about controlling manufacturing costs. Another query to be answered is the need for further safety studies, especially depending on the nanocellulose’s origin. Nonetheless, bacterial nanocellulose has already offered the pharmaceutical and cosmetic industry an opportunity, in the form of the development of high-tech bio platforms. Thus, in a general way, the scale-up of the nanocellulose production process and manufacturing, in order to obtain the optimal price-performance in its different applications remains a challenge as well. As part of the scientific advancements, there are several new concepts and methods of employing nanocellulose to deliver therapeutic molecules. The ability of nanocellulose to deliver biologics has not been explored much; therefore, some nanocellulose properties must be evaluated to choose the better type, since it will depend on the size and shape, charge, chemical composition and hydrophilicity of the molecule to be delivered. The subject is still new [134] and requires knowledge on the extraction and synthesis methods of the nanocellulose obtained, in order to choose the appropriate type to the delivery of biologics using nanocellulose.

## Figures and Tables

**Table 1:** The three types of nanocellulose.

Type of Nanocellulose	Related Terms	Typical Sources	Formation	Dimensions	Reference
Bacterial nanocellulose (BNC)	Bacterial cellulose; microbial cellulose; biocellulose	Low-molecular sugars and alcohols	Synthesized by several species of Acetobacteraceae	20–100 nm	[2,44]
Nanocrystalline cellulose (NCC)	Cellulose nanocrystal (CNC); nanocellulose whisker (CNW); rodlike cellulose microcrystals	Wood, cotton, hemp, flax, wheat straw, mulberry bark, ramie, tunicin and cellulose from algae and bacteria	Acid hydrolysis to remove amorphous cellulose	D *: 5–70 nm L *: 100–250 nm	[3]
Nanofibrillated cellulose (NFC)	Microfibrillated cellulose (MFC); cellulose nanofibers (CNF); nanofibrils; microfibrils	Wood, sugar beets, potatoes, hemp and flax	Delamination of wood pulp by high-pressure homogenization; microfluidization or grinding and/or following chemical or enzymatic treatments	D *: 5–60 nm L *: several μm	[49]

* D: diameter; L: length.

## Supplementary Materials

The following supporting information can be downloaded at: https://www.mdpi.com/article/10.3390/pharmaceutics16010145/s1, Table S1: patents separated by clusters after examination of documents obtained in WIPO, Espacenet and Lens.org databases, comprising the timeframe between 2011 and August 2023, with the search terms “nanofibrillar cellulose”, “nanofibrillated cellulose”, “cellulose nanofibers”, “microfibrillated cellulose”, “cellulose nanocrystals”, “nanocrystalline cellulose”, “nanocellulose”, “pharmaceutical”, “excipient” and “drug delivery” combined with Boolean operator and truncation symbol; Table S2: patents separated by clusters after examination of documents obtained in WIPO, Espacenet, and Lens.org databases, comprising the timeframe between 2011 and August 2023, with the search terms “bacterial nanocellulose”, “pharmaceutical”, “excipient”, and “drug delivery” combined with Boolean operator and truncation symbol.
