# Supplementary material for: Nanocellulose, the Green Biopolymer Trending in Pharmaceuticals: A Patent Review
Source: Pharmaceutics. 2024 Jan 21;16(1):145. doi: 10.3390/pharmaceutics16010145 (PMC10819157; doi:10.3390/pharmaceutics16010145)
Supplement: Supplementary file 1 [file pharmaceutics-16-00145-s001.zip › pharmaceutics-2815740-SI.pdf]

Review

Nanocellulose, the Green Biopolymer Trending in Pharmaceuticals: A Patent Review

Keth Ribeiro Garcia [1], Ruy Carlos Ruver Beck [1], Rosmary Nichele Brandalise [2], Venina dos Santos [2] and Letícia Scherer Koester [1,*]

[1] Programa de Pós-Graduação em Ciências Farmacêuticas, Faculdade de Farmácia, Universidade Federal do Rio Grande do Sul (UFRGS), Porto Alegre 90610-000, Brazil; k1rgarcia@gmail.com (K.R.G.); ruy.beck@ufrgs.br (R.C.R.B.)

[2] Programa de Pós-Graduação em Engenharia de Processos e Tecnologias, Universidade de Caxias do Sul (UCS), Caxias do Sul 95070-560, Brazil; rnbranda@ucs.br (R.N.B.); vsantos2@ucs.br (V.d.S.)

* Correspondence: leticia.koester@ufrgs.br; Tel.: +55-51-33085278

Supplementary material

Table S1. Patents separated by clusters after examination of documents obtained in WIPO, Espacenet, and Lens.org databases, comprising the timeframe between 2011 – Aug., 2023, with the search terms “nanofibrillar cellulose”, “nanofibrillated cellulose”, “cellulose nanofibers”, “microfibrillated cellulose”, “cellulose nanocrystals”, “nanocrystalline cellulose”, “nanocellulose”, “pharmaceutical”, “excipient”, and “drug delivery” combined with Boolean operator and truncation symbol.

Cluster	***Patent number***		
Design & Manufacturing	US 2018/0094081 A1	US 2020/0032454 A1	US 2023/0220118 A1
• Manufacture	US 10626191 B2	EP 3382095 A1	US 11572418 B2
	WO 2016/177395 A1	WO 2018/177878 A1	EP 3947533 B1
	US 10626191 B2	US 2019/0298838 A1	US 2023/0151545 A1
	WO 2019/238327 A1	WO 2016/109479 A1	US 11649296 B2
	US 2021/0130500 A1	US 2016/0184438 A1	US 11649333 B2
	EP 3581591 A1	EP 3212676 B1	EP 3533458 B1
	US 2021/0077403 A1	WO 2020/075057 A1	US 11602566 B2
	EP 3791864 A1	US 2019/0040581 A1	US 11634549 B2
	WO 2016/102767 A1	US 10883226 B2	US 11572450 B2
	US 2018/0179703 A1	WO 2017/134334 A1	US 11549085 B2
	US 10822743 B2	EP 3574143 B1	US 2022/0387955 A1
	WO 2016/193548 A1	US 2020/0023409 A1	WO 2022/249052 A1
	WO 2017/174874 A1	WO 2018/138702 A1	EP 4094827 A1
	EP 3228329 A1	WO 2020/058803 A1	US 2022/0389121 A1
	US 2019/0111175 A1	WO 2020/075056 A1	US 11440974 B2
	WO 2016/128620 A1	WO 2019/123405 A1	US 2022/0389261 A1
	US 2018/0021473 A1	WO 2019/073370 A1	US 11511015 B2
	US 10729804 B2	US 2021/0207324 A1	US 2022/0349651 A1
	US 2021/0205490 A1	US 2019/0234020 A1	EP 4083112 A1
	WO 2017/115018 A1	US 10927504 B2	US 2022/0275583 A1
	EP 3187195 A1	WO 2017/221137 A1	US 2022/0249378 A1
	EP 3187195 B1	EP 3475485 B1	US 2022/0233412 A1
	EP 3077592 B1	WO 2010/092239 A1	US 2022/0213298 A1
	WO 2015/082774 A1	US 9181653 B2	US 2022/0186049 A1
	EP 3077592 B2	US 2014/0182797 A1	US 2022/0185976 A1
	US 10214855 B2	US 2020/0086604 A1	US 2022/0177661 A1
	US 2016/0289894 A1	WO 2018/189698 A1	US 2022/0177660 A1
	US 2017/0190799 A1	WO 2019/171279 A1	EP 3228329 B1
	US 10113006 B2	WO 2020/102605 A1	WO 2022/097095 A1
	WO 2015/197906 A1	WO 2011/030170 A1	US 11324701 B2
	WO 2016/024046 A1	WO 2021/123499 A1	EP 3970945 A1
	EP 3350368 B1	US 10813377 B2	US 2022/0025147 A1
	US 2017/0218567 A1	CA 3003670 A1	US 2022/0018060 A1
	US 10570564 B2	US 2021/0045422 A1	
	WO 2017/046455 A1	WO 2017/075417 A1	
	RU 2719165 C1	US 2018/0319957 A1	
	WO 2017/103328 A1	CN 108430237 A	
	US 10640632 B2	CA 3003670 C	
	US 2018/0371211 A1	WO 2017/105337 A1	
	CN 103520739 A	US 2018/0291119 A1	
	US 9611586 B2	US 11084885 B2	
	US 2014/0323714 A1	US 2020/0157250 A1	
	EP 2782937 B1	WO 2018/231143 A1	
	US 10570564 B2	WO 2020/227816 A1	
	EP 3581590 A1	WO 2020/201627 A1	
	WO 2013/076376 A1	US 9970159 B2	
	US 10113005 B2	US 2018/0044856 A1	
	EP 2935446 B1	WO 2018/013034 A1	
	US 2015/0322170 A1	WO 2021/229406 A1	
	WO 2014/096547 A1	US 11174324 B2	

• Process	US 10625232 B2<br>WO 2016/102766 A1<br>US 2017/0348662 A1<br>EP 3237108 B1<br>US 2021/0229057 A1<br>US 2017/0348663 A1<br>WO 2016/102764 A1<br>US 10981135 B2<br>EP 3237107 B1<br>US 2020/0078305 A1<br>WO 2018/109281 A1<br>WO 2016/102765 A1<br>US 2017/0368211 A1<br>US 10322196 B2<br>EP 3237025 B1<br>EP 3335695 A1<br>EP 3335695 B1	US 2021/0127663 A1<br>WO 2018/108341 A1<br>EP 3335696 B1<br>EP 3335696 A1<br>WO 2018/109282 A1<br>US 2019/0343889 A1<br>WO 2010/102802 A1<br>US 10017583 B2<br>US 2016/0130368 A1<br>WO 2014/195971 A1<br>EP 2812483 B2<br>US 2014/0374045 A1<br>EP 2812483 B1<br>US 2016/0186376 A1<br>US 9315942 B2<br>WO 2013/117823 A1<br>US 9725849 B2	WO 2019/116245 A1<br>WO 2012/152997 A1<br>US 2014/0130710 A1<br>EP 4201963 A1<br>WO 2023/084383 A1<br>EP 3331939 B1<br>US 11555186 B2<br>EP 4083113 A1<br>US 2022/0325003 A1<br>US 11421118 B2<br>WO 2022/006644 A1
Cell culture systems and cell technology	US 2020/0385660 A1<br>EP 3750982 A1<br>US 2021/0214510 A1<br>EP 3572434 A1<br>EP 3669651 A1<br>US 2020/0199536 A1<br>WO 2020/260741 A1<br>WO 2021/127373 A1<br>EP 3234600 B1<br>US 2021/0190765 A1<br>US 10976308 B2<br>WO 2016/097490 A1<br>US 2018/0024121 A1<br>EP 2900806 B1<br>WO 2014/049204 A1<br>EP 3699264 A1<br>US 2015/0267164 A1<br>US 9593304 B2	US 2020/0164103 A1<br>WO 2016/100856 A1<br>EP 3233493 B1<br>US 2020/0206385 A1<br>US 2017/0368225 A1<br>US 10675379 B2<br>EP 3418377 A1<br>WO 2018/234634 A1<br>US 2020/0115678 A1<br>CA 3140249 A1<br>US 11186736 B2<br>US 2021/0179871 A1<br>EP 2975115 A1<br>EP 3669651 B1<br>WO 2023/113685 A1<br>EP 3699264 B1<br>US 2023/0082321 A1<br>EP 4148071 A1	WO 2023/012333 A2<br>EP 4119657 A1<br>EP 4082587 A1<br>EP 3684433 B1<br>WO 2022/207889 A1<br>WO 2022/144489 A1<br>EP 4023744 A1<br>US 2022/0213432 A1<br>US 2022/0214329 A1<br>EP 3967748 A1<br>US 11332714 B2<br>AU 2020/312637 A1<br>AU 2020/313511 A1<br>CN 114127279 A<br>CN 114080242 A<br>MX 2021015757 A<br>MX 2021015722 A
Drug delivery			
• Injectable	CA 3101156 A1<br>US 2021/0085602 A1<br>JP 2021046393 A<br>EP 3791858 A1	WO 2012/056111 A2<br>EP 2632493 B1<br>US 2013/0330379 A1<br>US 9631177 B2<br>US 2022/0257753 A1	
• Oral	WO 2021/071974 A1<br>CN 112336741 A<br>US 2021/0030782 A1<br>WO 2019/094769 A1	WO 2021/116917 A1<br>WO 2018/009139 A1<br>WO 2013/009253 A1<br>CN 115400101 A<br>CN 114732801 A	
• Buccal	WO 2018/104971 A1<br>WO 2018/152627 A1<br>US 2020/0281868 A1<br>US 11478435 B2		
• Topical	WO 2020/031186 A1<br>US 9816230 B2<br>US 2016/0186377 A1<br>WO 2018/009112 A1	WO 2018/009113 A1<br>US 2020/0188538 A1<br>WO 2017/117650 A1<br>WO 2020/227814 A1	
• Transdermal	WO 2021/108722 A1<br>US 2020/0375878 A1<br>EP 3730122 A2		

• Matrix	WO 2013/072563 A1<br>US 2014/0322327 A1<br>US 2021/0015121 A1<br>WO 2019/108887 A1<br>US 2020/0254046 A1<br>WO 2018/015175 A9<br>WO 2018/015175 A1<br>WO 2023/009976 A1<br>US 2023/0028624 A1<br>US 2023/0017712 A1<br>US 11389537 B2	
Wound healing	EP 3771470 A1<br>US 2021/0030919 A1<br>WO 2019/166606 A1<br>EP 3533458 A1<br>US 2021/0170041 A1<br>EP 3816338 A1<br>US 2021/0128365 A1<br>WO 2018/109275 A1<br>EP 3335740 A1<br>US 2019/0336643 A1<br>US 2014/0213764 A1	US 2013/0330417 A1<br>WO 2017/103263 A1<br>EP 3181152 A1<br>US 2016/0325011 A1<br>EP 3088008 A1<br>EP 3402462 B1<br>WO 2017/122224 A1<br>US 2019/0015468 A1<br>US 10426809 B2<br>WO 2018/130390 A1<br>US 11173228 B2<br>WO 2023/118589 A1<br>US 2023/0181791 A1<br>US 11534526 B2
Tissue engineering	WO 2020/249814 A1<br>US 2021/0069964 A1<br>US 2021/0138113 A1<br>WO 2019/211854 A1<br>WO 2020/249814 A1<br>WO 2019/122351 A1<br>US 2019/0015550 A1<br>US 11103617 B1<br>US 11167062 B2<br>US 11713444 B2	US 2023/0226098 A1<br>WO 2023/133517 A1<br>WO 2023/118966 A1<br>US 11602579 B2<br>US 2022/0249738 A1<br>US 2022/0145259 A1<br>WO 2022/003203 A1<br>EP 3932437 A1

Table S2. Patents separated by clusters after examination of documents obtained in WIPO, Espacenet, and Lens.org databases, comprising the timeframe between 2011 - Aug., 2023, with the search terms "bacterial nanocellulose", "pharmaceutical", "excipient", and "drug delivery" combined with Boolean operator and truncation symbol.

Cluster	*Patent number*	
Design & Manufacturing	WO 2020/136629 A1	US 2019/0060205 A1
• Manufacture	US 10975242 B2	WO 2012/106707 A3
	US 10982387 B2	US 2013/0309295 A1
	JP 2021065224 A	WO 2012/106707 A2
	CN 109223727 A	KR 20200085090 A
	WO 2021/009021 A1	US 2021/0015966 A1
	WO 2021/009038 A1	WO 2023/108017 A2
		WO 2023/070028 A1
		WO 2023/048674 A1
		WO 2022/200631 A1
• Process	US 10870950 B2	
	WO 2017/165465 A1	
	US 2021/0071366 A1	
	US 2019/0093288 A1	
	WO 2023/089010 A1	
Drug delivery		
• Intraocular	US 2013/0011385 A1	
	US 8940337 B2	
	WO 2011/079380 A1	
• Oral	CN 103191441 A	
	CN 102743356 A	
• Topical	CN 210673549 U	US 2018/0303726 A1
	US 2011/0039744 A1	US 2020/0364264 A1
	WO 2011/019876 A2	EP 3812494 A1
	US 8097574 B2	US 2021/0140100 A1
	US 10736823 B2	US 2011/0286948 A1
	EP 3380072 B1	US 8772003 B2
	WO 2017/089005 A1	
• Transdermal	CN 106074458 A	
• Matrix	US 2012/0308649 A1	
	US 8871743 B2	
	WO 2020/058836 A1	
Wound healing	CN 111265709 A	US 9314531 B2
	US 9211256 B2	US 2012/0231038 A1
	US 2016/0074520 A1	
Tissue engineering	US 2018/0298370 A1	CN 105031722 A
	WO 2016/174104 A1	US 2016/0354406 A1
	RO 130767 A0	US 10130653 B2
	US 10767205 B2	WO 2021/245324 A1
